# Reduced Toxicity of Shiga Toxin (Stx) Type 2c in Mice Compared to Stx2d Is Associated with Instability of Stx2c Holotoxin

**DOI:** 10.3390/toxins7062306

**Published:** 2015-06-23

**Authors:** Joshua C. Bunger, Angela R. Melton-Celsa, Ernest L. Maynard, Alison D. O’Brien

**Affiliations:** 1 Department of Microbiology and Immunology, Uniformed Services University of the Health Sciences, Bethesda, MD 20814-4799, USA; E-Mails: jbunger@gmail.com (J.C.B.); angela.melton-celsa.ctr@usuhs.edu (A.R.M.-C.); 2 Department of Biochemistry and Molecular Biology, Uniformed Services University of the Health Sciences, Bethesda, MD 20814-4799, USA; E-Mail: ernie.maynard@gmail.com; 3 Current Address: National Center for Biotechnology Information, National Library of Medicine, National Institutes of Health, Bethesda, MD 20894, USA

**Keywords:** Shiga toxin, Stx2, Stx2c, Stx2d, STEC

## Abstract

Shiga toxin (Stx) is an AB_5_ ribotoxin made by Stx-producing *Escherichia coli* (STEC). These organisms cause diarrhea, hemorrhagic colitis and the hemolytic uremic syndrome. STEC make two types of Stxs, Stx1 and/or Stx2. Stx2 has one prototype (a) and six subtypes (b–g), but only STEC that make Stx2a, and/or Stx2c, or Stx2d are associated with severe disease. However, Stx2c is about 10-fold less toxic than Stx2d *in vivo* despite only two amino acid differences in the A subunit at positions 291 and 297. We made mutations at these two sites to create intermediate toxins between Stx2c and Stx2d, and determined the 50% cytotoxic dose on Vero cells before and after heat treatment, and the 50% lethal dose in mice of the toxins. We found that serine 291 was associated with increased toxicity *in vivo* and that either amino acid change from that in Stx2c to that in Stx2d increased heat stability. We also assessed the secondary structure of Stx2c and Stx2d by circular dichroism (CD) spectroscopy. The CD studies suggest that Stx2c has a less-ordered secondary structure than Stx2d. We conclude that both amino acids at positions 291 and 297 in Stx2c contribute to its decreased stability and *in vivo* toxicity compared to Stx2d.

## 1. Introduction

Shiga toxin (Stx)-producing *Escherichia coli* (STEC) have the capacity to produce at least one of the two types of Stxs, Stx type 1 (Stx1) or Stx type 2 (Stx2). Stx1 and Stx2 are highly homologous and have a similar mode of action [1]. STEC strains can cause severe foodborne illnesses, such as hemorrhagic colitis (HC) and the potentially deadly hemolytic uremic syndrome (HUS) [2]. Moreover, the HC and HUS can be directly attributed to the presence of the Stxs [3]. Approximately half of individuals infected with an STEC strain will naturally self-resolve without symptoms and the other half will develop HC [4,5,6]. Among those individuals who present with HC, up to one in ten will progress to the HUS. HUS is characterized by microangiopathic hemolytic anemia, thrombocytopenia, renal damage, occasional neurological complications, and a fatality rate of up to 5% [7,8]. There is currently no Food and Drug Administration (FDA)-approved vaccine to prevent STEC infections or disease. Moreover, antibiotic therapy is contraindicated as certain antibiotics increase the amount of Stx produced by STEC and are linked in some studies to an increased incidence of the HUS [9,10,11,12].

The Stxs are AB_5_ ribosome-inactivating holotoxins with one enzymatically-active A subunit, a small portion of which is threaded through five binding B subunits that form a pentameric ring [13,14]. Stxs bind to glycosphingolipids (GSLs) expressed on the surface of cells, typically globotriaosylceramide (Gb3/CD77) [15,16,17,18]. While Stx receptor specificity and attachment to cells are dictated by the B subunit, the *C*-terminal portion of the Stx2 A subunit that extends through the B pentamer can reduce the overall capacity of the holotoxin to affix to Gb3 [13,19,20,21]. Toxin is internalized via endocytosis and is trafficked to the Golgi, where the A subunit is cleaved by furin into an A_1_ moiety (27,300 kDa) and an approximately 5 kDa A_2_ peptide. The A_2_ peptide remains threaded through the B pentamer, and the A_1_ moiety remains attached to A_2_B_5_ by a disulfide bond between A_1_ and A_2_. The holotoxin transits to the endoplasmic reticulum where the A_1_ subunit is released into the cytoplasm. In the cytoplasm, the A_1_ subunit disables the host 60S ribosomal subunit by cleaving a single residue of the 28S ribosomal RNA (reviewed in [22]). This cleavage event leads to permanent inhibition of host cell protein synthesis and ultimately cell death (reviewed in [23]).

**Figure 1:**
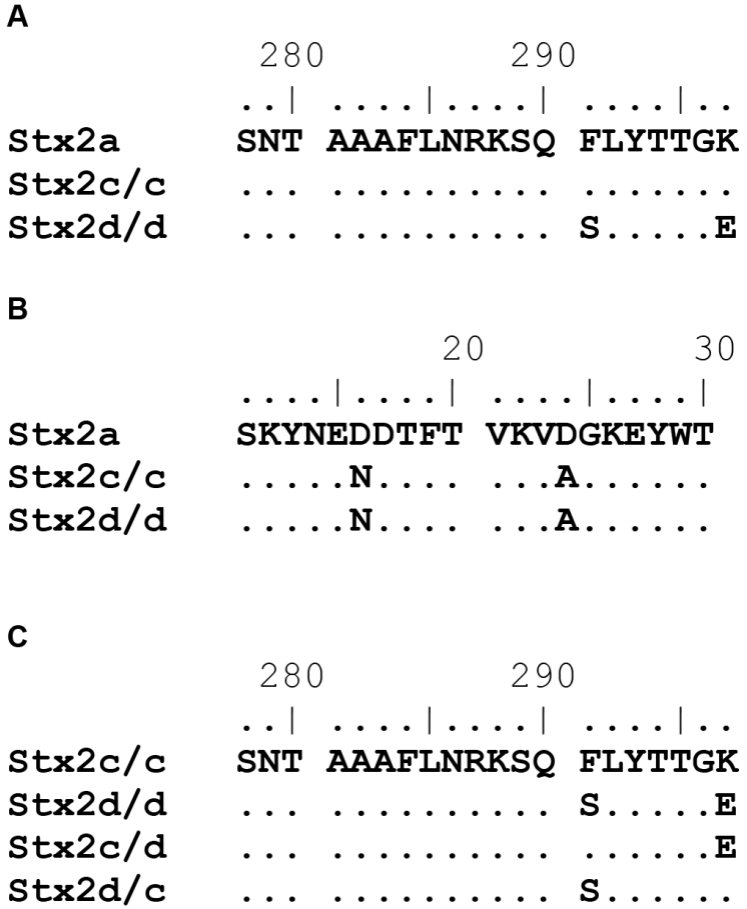
Partial amino acid sequence alignment of the *C*-terminus of the A subunit and B subunit from Stx2a, Stx2c/c, Stx2d/d, Stx2c/d, and Stx2d/c. Amino acid sequence alignment of a portion of the *C*-terminus of the A subunit that shows the F291S and K297E changes in Stx2d/d with Stx2a as the reference sequence (**A**); Amino acid sequence alignment of the B subunit that shows the D16N and D24A changes in Stx2c/c and Stx2d/d with Stx2a as the reference sequence (**B**); Amino acid sequence alignment that shows the sequence in the intermediate toxin A subunits Stx2c/d and Stx2d/c with Stx2c/c as the reference sequence and Stx2d/d for comparison (the B subunits were not altered) (**C**). Sequence alignments were compiled in BioEdit.

The Stx2 serogroup has one prototype toxin and six subtype toxins: the prototype Stx2a, and subtypes Stx2b, Stx2c, Stx2d, Stx2e, Stx2f, and Stx2g [24]. STEC outbreak isolates that make Stx2a, Stx2c, and/or Stx2d are more commonly associated with severe disease than those strains that make Stx1 or Stx1 and Stx2 [25,26,27,28]. Stx2a, Stx2c, and Stx2d are quite similar in amino acid sequence (~98.9%). Stx2a and Stx2c share two amino acids in the carboxyl terminus of the A_2_ peptide that are different from those of Stx2d at positions 291 and 297 (Figure 1A). Stx2c and Stx2d share two amino acids in the B subunit that are different from those of Stx2a at positions 16 and 24 (Figure 1B). Differences in observed Vero cell toxicity between Stx2a compared to Stx2c and Stx2d can be explained by the two amino acid differences in the B subunit [29]. While Stx2c and Stx2d have similar toxicities for Vero cells, most likely due to identical B subunits, they have different toxicities for mice. In fact, the 50% lethal dose (LD_50_) of Stx2d is closer to that of Stx2a than Stx2c [20,29,30]. The lower LD_50_ of Stx2d compared to Stx2c is likely due, at least in part, to the activation of Stx2d by elastase [20]. In this study, we further investigated whether the difference in toxicity between Stx2c and Stx2d is due to one and/or the other amino acids at positions 291 and 297 in the A subunit even in the absence of activation by mucus. We found that both amino acids contribute to the differential toxicity of Stx2c and Stx2d.

## 2. Results and Discussion

### 2.1. Results

#### 2.1.1. Assessment of Purified Toxins *in Vitro* and *in Vivo*

A comparison of the A and B subunit amino acid alignments between Stx2c/c and Stx2d/d reveals only two amino acid differences among the toxins in the A_2_ peptide of the molecules (Figure 1A) and no differences in the B subunit (Figure 1B). The binding and toxicity of Stxs to cells is directly related to the B subunit ([31,32], reviewed in [23]), but some studies have shown that the A_2_ subunit, specifically the C terminus, can alter the binding affinity of the toxin to Gb3 [19,20]. Therefore, we generated toxins that were intermediates in the A_2_ subunit between Stx2c/c and Stx2d/d (Figure 1C) to determine the impact of each amino acid difference with respect to biological activity. The naming convention for our toxins indicates which parental amino acid is found in positions 291/297. We then expressed and purified the two parental and two intermediate toxins, Stx2c/c, Stx2d/d, Stx2c/d, and Stx2d/c.

Next, we tested whether these amino acid changes altered the toxicity of the molecules *in vitro* compared to the parent toxins. We found that the specific Vero cell cytotoxic activities of Stx2c/c, Stx2d/d, and Stx2d/c were highly similar with a specific activity between log 7.0–7.4 CD_50_/mg. Toxin Stx2c/d showed a significantly reduced specific activity of about one log less than the others (Table 1). We then evaluated the impact of the amino acid changes on the *in vivo* activity of the toxins after IP injection into mice. We found the LD_50_ of Stx2d/d and Stx2d/c to be approximately 10X lower than Stx2c/c and Stx2c/d. Specifically, toxins that had a serine at position 291 had lower LD_50_ values. From these experiments we concluded that Stx2c/d exhibited reduced toxicity for Vero cells *in vitro* compared to the other toxins and that the presence of serine at position 291 was positively correlated with toxicity *in vivo* compared to phenylalanine at position 291. We next tested the capacity of each toxin to bind Gb3.

**Table 1:** Cytotoxicity and LD_50_ values for the toxins.

Toxin	Amino Acids at Position 291 and 297, Respectively	Log Specific Activity, CD_50_/mg	CD_50_ 95% Confidence Interval	LD_50_, ng	LD_50_ 95% Confidence Interval, ng
Stx2c/c	F, K	7.0	6.5–7.5	14	5.1–38
Stx2d/d	S, E	7.0	5.5–7.0	1.8	0.94–3.0
Stx2c/d	F, E	6.3 *	7.0–7.9	24	12–49
Stx2d/c	S, K	7.4	6.8–7.1	2.5	1.5–3.9

* The specific activity of Stx2c/d was significantly reduced compared to the three other toxins, *p* ≤ 0.025 as determined by analysis of variance followed by unpaired *t* tests.

#### 2.1.2. Binding of Toxins to Purified Gb3

**Figure 2:**
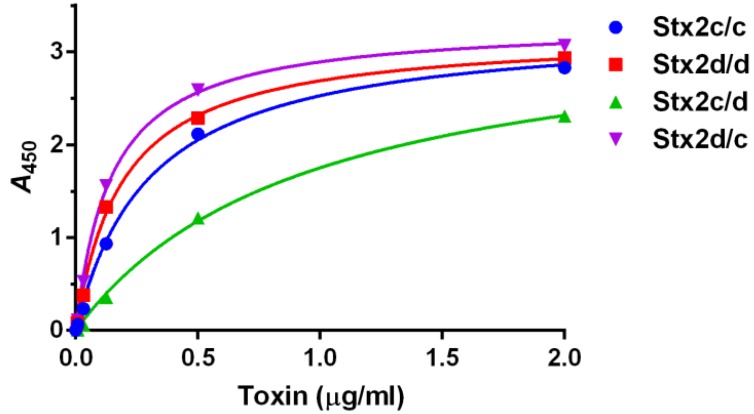
Binding of toxins to Gb3. An ELISA was used to assess binding of each toxin to purified Gb3. Mean and standard deviation values were plotted from triplicate values. The nonlinear curve was approximated by “One Site–Specific Binding” within GraphPad Prism (see Materials and Methods) and the *r*^2^ > 0.99 for all toxins curves. An F test to compare the *K_d_* values indicated that they were different, *p* < 0.0001, and pairwise comparisons gave a value of *p* ≤ 0.0001. The apparent *K_d_* values (rounded to the nearest 0.01) for Stx2c/c, Stx2d/d, Stx2c/d, and Stx2d/c, were 0.30 µg/mL with a 95% CI of 0.26–0.35 µg/mL, 0.19 µg/mL with a 95% CI of 0.17–0.21 µg/mL, 0.95 µg/mL with a 95% CI of 0.82–1.1 µg/mL, and 0.15 µg/mL with a 95% CI of 0.13–0.16 µg/mL, respectively. The B_max_ values were similar for each toxin and ranged from 3.2 to 3.4.

Since Stx2c/c showed reduced toxicity compared to Stx2d/d *in vivo* but not *in vitro* (Table 1), we next sought to determine if there were any Gb3-binding differences among the toxins or the intermediates that might help explain that difference. We therefore estimated the *K_d_* of each toxin for Gb3 in an ELISA. We found the differences among the *K_d_* values as determined in the Gb3-binding ELISA (apparent K*_d_*) for the toxins and intermediates to be statistically significant with a *p* value < 0.0001, although there was no difference in B_max_. The smallest apparent *K_d_* value was measured for Stx2d/c and the largest apparent *K_d_* value from Stx2c/d, (Figure 2). These results indicated that a change from glutamic acid to lysine at position 297 in the presence of serine at position 291 resulted in an enhancement of binding to purified Gb3 since Stx2d/c had a smaller apparent *K_d_* than Stx2c/c. However, the alteration of the Stx2c/c terminal amino acid from lysine to glutamic acid in Stx2c/d led to a statistically significant decrease in binding to purified Gb3 with an increase in apparent *K_d_* observed. Because Stx2c/d had the largest *K_d_* for Gb3 and, therefore, bound less tightly to Gb3 than the other three toxins, we hypothesized that (1) the combination of phenylalanine/glutamic acid at positions 291/297 is responsible for the elevated *K_d_* of Stx2c/d; (2) the reduced binding of Stx2c/d to Gb3 is tied to its reduced *in vitro* and *in vivo* toxicity; and, (3) the reason for the reduced binding of Stx2c/d to Gb3 is that that toxin is less stable than the other three toxins. We further hypothesized that the presence of a serine at position 291 rather than phenylalanine may allow for a more stable holotoxin as demonstrated by the lower *K_d_* values measured for Stx2d/d and Stx2d/c. Therefore, to test stability of the four toxins, we measured the thermostability of the toxins as described in the next section.

#### 2.1.3. Thermostability of Toxins

**Figure 3:**
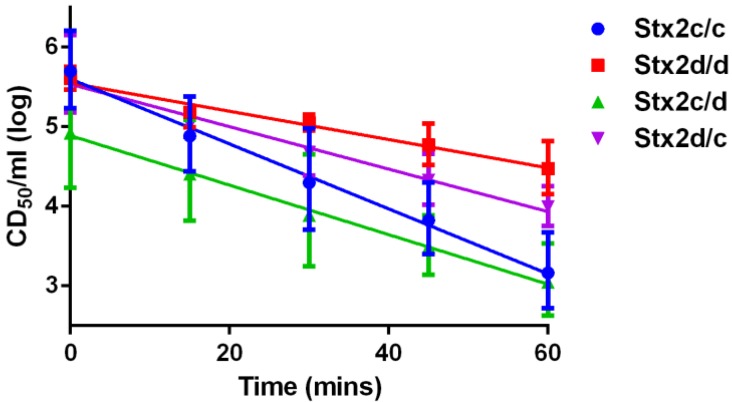
Thermostability assay. Toxins were incubated at 70 °C for the time indicated by the points on each line and then placed on Vero cells. Data are reported as the geometric mean with 95% confidence intervals from triplicate samples. Linear approximations were done with GraphPad Prism with a resultant *r*^2^ ≥ 0.92 for all toxins. Analysis of covariance showed these approximated slopes to be statistically significantly different with a *p* < 0.0001. Statistical differences of pairwise comparisons: Stx2c/c versus Stx2c/d, *p* = 0.011; Stx2c/d *versus* Stx2d/c, *p* = 0.166; and all other comparisons, *p* < 0.001.

We assessed the thermostability of each toxin by heat treatment followed by assessment of cytotoxicity on Vero cells. We found that the cytotoxicity of each toxin decreased over time after heat treatment and could be plotted with a negative linear slope with an *r*^2^ ≥ 0.92 for all toxins (Figure 3). We then did an analysis of covariance (ANCOVA) of the estimated slopes to compare the decrease in toxicity of each toxin. The slopes of the lines were statistically different with a *p* value < 0.0001. Next, six pairwise comparisons between the toxins/intermediates were done to estimate the similarity of the estimated slopes of the cytotoxicity lines after heat treatment of the toxins over time. Two slope comparisons were not statistically significant after the Bonferroni correction: those of Stx2c/c and Stx2c/d with a *p* value = 0.011 and Stx2c/d and Stx2d/c with a *p* value = 0.166. All other pairwise comparisons had a *p* value < 0.001. Thus, our thermostability assay revealed that Stx2d/d remained the most stable over 1 h at 70 °C and Stx2c/c was the least stable. Furthermore, Stx2c/d and Stx2d/c showed intermediate stabilities and were not statistically different from each other. These results indicate that both serine at position 291 and glutamic acid at position 297 play a role in increased thermostability.

#### 2.1.4. Secondary Structure of Stx2c and Stx2d

**Figure 4:**
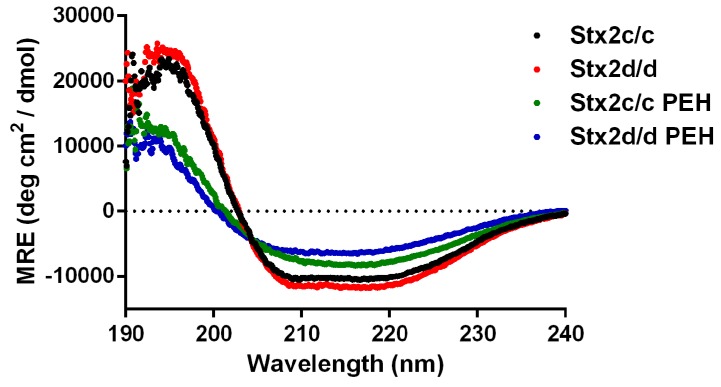
Circular dichroism spectra of Stx2c/c and Stx2d/d. Mean residual ellipticitities (MRE) are plotted as a function of wavelength. Spectra of native Stx2c/c and Stx2d/d were acquired at 20 °C. Post exposure to heat (PEH) spectra were acquired at 40 °C. Secondary structural analyses of both native and PEH toxins were done as described in the Experimental section, and the results are presented in Table 2.

Our thermostability experiments indicated that Stx2d/d was more thermostable than Stx2c/c. The thermostability assay is a good marker for overall protein stability, but it ultimately is an indirect assessment of the structure of these toxins. Therefore, we used CD spectroscopy to probe secondary structural differences between Stx2c/c and Stx2d/d. The spectra of Stx2c/c and Stx2d/d prior to heat treatment were similar (Figure 4). Spectral deconvolution of the CD spectra yielded similar results in percent alpha helix and beta strand, but showed a striking difference in the percent of turns (Table 2). The remainder of predicted secondary structure is defined as “unordered”, and Stx2c/c showed a larger percentage for that parameter than did Stx2d/d (Table 2). In summary, although helical and strand secondary structure percentages were similar for Stx2c/c and Stx2d/d, Stx2d/d had a significantly higher level of turns (20%) compared to Stx2c/c (11%), a finding that suggests a higher level of structural order in Stx2d/d than Stx2c/c. The CD spectra of Stx2c/c and Stx2d/d post-exposure to heat (PEH) indicate that the heating process caused an irreversible structural change in both toxins (Figure 4). In addition, deconvolution of the CD spectra revealed that Stx2c/c and Stx2d/d shared similar helix, strand, and turn percentages after heat treatment. In particular, the percent turn and unordered structure were almost identical for Stx2d/d and Stx2c/c (Table 2). Overall the data in Figure 4 and Table 2 suggest that heat treatment converts both toxins into conformations with similar secondary structure profiles and highlight the link between Stx2 structure and toxicity. Furthermore, the data in Table 2 provide insight into structural differences that may contribute to the increased toxicity of Stx2d/d compared to Stx2c/c before heat treatment; specifically, a lower percentage of unordered structure.

**Table 2:** Secondary structural analyses of Stx2c/c and Stx2d/d before and post exposure to heat (PEH).

Toxin	Helix, % (SD *)	Strand, % (SD)	Turns, % (SD)	Unordered, % (SD)
**Stx2c/c**	31.2 (0.9)	26.3 (1.6)	10.6 (0.3)	31.9 (1.0)
**Stx2d/d**	31.0 (1.0)	25.3 (1.5)	20.0 (0.7)	23.6 (0.8)
**Stx2c/c PEH**	18.4 (0.5)	33.4 (1.3)	11.6 (0.3)	36.6 (1.0)
**Stx2d/d PEH**	12.5 (0.4)	37.9 (2.0)	11.8 (0.4)	37.8 (1.2)

* SD: standard deviation. The errors are the normalized root-mean-square deviation of 15 of the best-fit solutions.

Thus, the different ordered states of Stx2c/c and Stx2d/d may account for the differences observed in mouse toxicity but is not a factor in the *in vitro* environment of cell cultures. Together, these results showed that Stx2c/c and Stx2d/d have similar but detectibly different structures and that these structural differences are dependent on positions 291 and 297 of the A subunit.

### 2.2. Discussion

Stx2c/c and Stx2d/d are differentiated by only two amino acid differences located at the *C*-terminus of the A_2_ peptide that is found on the Gb3-binding face of the holotoxin, yet, both toxins are found in strains that cause the HUS [28,33]. From these observations, one might surmise that the *in vitro* and *in vivo* toxicity of these toxins would be similar. However, we, and others, have found that the specific activities are the same for Stx2c/c and Stx2d/d on Vero cells, but that the toxicity values *in vivo* are significantly different [29,30]. In this report, we linked the decreased toxicity of Stx2c/c compared to Stx2d/d in mice to the two amino acid differences between the toxins in the A_2_ peptide of the molecules, at amino acids 291 and 297. We also showed that these two amino acid differences result in decreased thermal stability of Stx2c/c compared to Stx2d/d.

**Figure 5:**
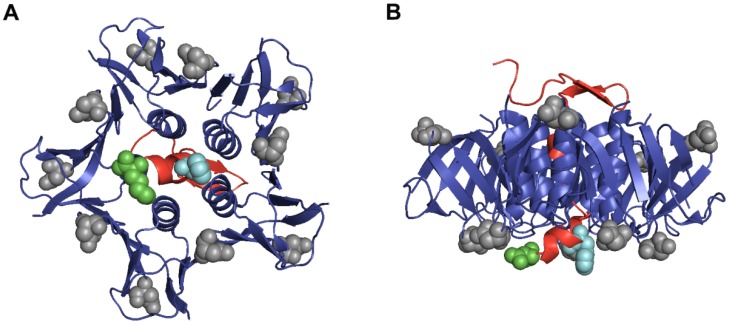
Crystal structure of A_2_ peptide and B subunits from Stx2a. A ribbon representation of the crystal structure of the A_2_ peptide shows the relative locations of the amino acids at positions 291 and 297 in comparison to positions 16 and 24 from the B subunits. The A_2_ peptide is colored red with a space-filled model of phenylalanine at position 291 depicted in teal and lysine at position 297 highlighted in green. The B subunits are colored blue with a space-filled model of aspartic acid at positions 16 and 24 highlighted in grey. Part (**A**) shows the binding face of Stx2a; Part (**B**) shows a horizontal view of the toxin with the binding face at the bottom. The PDB accession number for Stx2a is 1R4P. In this representation, the A_1_ portion of the structure is not shown.

Crystal structure analysis of Stx2a predicts that the *C*-terminus of the A_2_ peptide may partially block one of the binding sites of a B subunit for Gb3 (Figure 5A,B) [13]. In addition, we have shown that for Stx2d/d removal of the two *C*-terminal amino acids from A_2_ increases its toxicity and capacity to bind Gb3 [19,20]. After making intermediate toxins between Stx2c/c and Stx2d/d, we found that phenylalanine at 291 when present in conjunction with glutamic acid at position 297 (Stx2c/d) was the least toxic protein *in vitro* and *in vivo.* We speculate that this combination of amino acids may represent a relatively unstable molecule with a reduced capacity to form holotoxin. However, serine at position 291 in wild-type Stx2d/d or the chimeric protein Stx2d/c was positively correlated with toxicity *in vivo* regardless of the amino acid at position 297, a finding that highlights the importance of the amino acid at position 291.

The positions of amino acids 291 and 297 are located at the binding face of the B pentamer. Amino acid changes in the A_2_ subunit near the binding face of the B subunits can alter the activity of Stx2d for Vero cells and the interactions with Gb3 [19,20]. We found that Stx2d/d, which has a serine at position 291, did not have a reduction in binding to Gb3 if the amino acid at 297 was changed from glutamic acid to lysine. Alternatively, Stx2c/c, with phenylalanine at position 291, had a reduction in binding to Gb3 when the amino acid at 297 was changed from lysine to glutamic acid. These findings reinforced our *in vitro* toxicity data, as well as support the association between toxin binding capacities and cytotoxicity. Taken together, our data revealed that Stx2 toxins with serine at position 291 (Stx2d/d and Stx2d/c) had similar Gb3 binding capacity, cytotoxicity, and *in vivo* toxicity regardless of whether position 297 contained glutamic acid or lysine. We further speculate that the reason that Stx2a is more toxic to mice than Stx2c, even though Stx2a has the same 291/297 amino acid combination as Stx2c, is that the two amino acid changes in the B subunit at positions 16 and 24 compensate in some way for the phenylalanine and lysine in the A_2_ peptide.

Our toxins showed distinct patterns of activity. For example, Stx2c/c and Stx2d/d exhibited similar activity *in vitro*, but Stx2d/d was more toxic than Stx2c/c in mice. The thermostability assay gave us a way to indirectly assess the stability of each toxin. Of our four toxins tested, we found that Stx2d/d was the more thermostable toxin and that Stx2c/c was the least thermostable toxin. To our surprise, a change of either amino acid at position 291 or position 297 from Stx2d/d to Stx2c/c gave a statistically significant increase in thermostability compared to Stx2c/c alone. We had predicted that Stx2c/d would be the least thermostable of the four toxins given the Gb3 binding, cytotoxicity, and *in vivo* results, but we found that the thermostability of Stx2c/d was not statistically different from Stx2c/c after the Bonferroni correction. While the Bonferroni correction may be an overly conservative approach in this pairwise comparison we ultimately conclude that the increased thermostability of Stx2d/d compared to Stx2c/c is responsible for the increased toxicity observed *in vivo*.

CD spectroscopy was used to compare the secondary structures of Stx2c/c and Stx2d/d before and after exposure to heat. Before heat treatment, we found the spectral curves of Stx2c/c and Stx2d/d were similar, a result that was not surprising due to the near sequence identity of the toxins. However, deconvolution of their CD spectra showed that Stx2d/d contained a larger percentage of defined beta turns and thus displayed a more ordered state than Stx2c/c (Table 2). A large majority of beta turns from Stx2a are located in the B subunit (Figure 5A,B), and we hypothesize that the A_2_ subunit portion threaded in between or near the B pentamer may affect the secondary structure of the B subunits. After heat treatment, we observed irreversible structural changes in the CD spectra of Stx2c/c and Stx2d/d (Figure 4). Moreover, spectral deconvolution revealed a decrease in alpha helix percentage, an increase in beta strand percentage, and an increase in the unordered states of each toxin (Table 2).

We speculate that the enhanced toxicity of Stx2d/d compared to Stx2c/c in mice could be explained by the more highly ordered structural state of Stx2d/d. Furthermore, the beta turn percentages from Stx2d/d PEH resemble the beta turn percentages of native Stx2c/c and provide a structural explanation for the observed decrease in cytotoxicity following heat treatment. We propose the larger overall percentage of ordered secondary structure in Stx2d/d compared to Stx2c/c as the molecular basis for increased *in vivo* toxicity of Stx2d/d compared to Stx2c/c. Given the large number of beta turns in the crystal structure of the B subunit, we infer that this increased ordered state of Stx2d/d is due to the interactions between the A_2_ subunit and the B subunits. Such interactions between the A_2_ peptide and the B subunit are also indicated by the fact that Stx2c/c and Stx2d/d, which have the same B subunit, interacted differently with Gb3. Taken together, we showed that complex interactions occur between the A_2_ subunit and B subunits of Stx2c/c and Stx2d/d and that these interactions are separate from the capacity for toxin to bind Gb3. Lastly, these interactions are necessary for increased toxicity *in vivo*, but not *in vitro*.

## 3. Experimental Section

### 3.1. Toxins and Cell Culture

Stx2c/c, Stx2d/d, Stx2c/d, and Stx2d/c were purified from DH5α transformed with plasmid pCKS174 (stx_2c_ from E32511), pSQ543 (stx_2d2_ from B2F1), pAM1451, or pAM3472, respectively as described previously [33] except that the gel filtration column was not used. Briefly, cell-associated toxin was purified by immunoaffinity chromatography over an AminoLink Plus Immobilization column (Thermo Fisher Scientific, Rockford, IL, USA) to which 10 mg of polyclonal rabbit anti-Stx2a [20] had been bound. Toxin samples were subjected to sodium dodecyl sulfate polyacrylamide gel electrophoresis (SDS-PAGE), and the resultant gels were stained with Oriole fluorescent gel stain (Bio-Rad, Hercules, CA, USA) to assess toxin purity. Toxin purity was calculated based on the integrated density of the A and B subunits relative to the integrated density of the entire lane. Toxin concentrations were determined by multiplication of toxin purity by total protein concentration determined by the bicinchroninic acid assay value (Thermo Fisher Scientific). Purified toxin samples were dialyzed in 1X phosphate-buffered saline (PBS) with Slide-A-Lyser dialysis cassettes (10K MWCO; Thermo Fisher Scientific) and stored in 1X PBS at 4 °C. Vero cells (CCL-81, ATCC, Manassas, VA, USA) were maintained in Eagle’s minimal essential medium (EMEM) supplemented with 10% heat-inactivated fetal bovine serum (FBS), gentamicin (100 µg/mL), penicillin (10 U/mL), and streptomycin (10 µg/mL). Cells were grown at 37 °C in a 5% CO2 atmosphere.

### 3.2. Cytotoxicity Assay

Cytotoxicity assays were done on Vero cells. The 50% cytotoxic dose (CD_50_) was determined according to the method of Gentry and Dalrymple [34] as modified by Lindgren *et al.* [28]. Briefly, 10,000 Vero cells were seeded into each well of a 96-well plate, and plates were incubated for 24 h at 37 °C in 5% CO_2_. Cells were then overlaid with toxin diluted in medium or medium only and allowed to incubate for an additional 48 h. The cells were then fixed in 10% formalin, stained with a solution of 0.13% crystal violet and 5% ethanol in water, and the A630 measured as an estimate of the density of cells that remained in the wells. The reciprocal of the toxin dilution that caused 50% cell death was reported as the CD_50_ value.

### 3.3. Animal Research Ethics Statement

All animal studies were approved by the Institutional Animal Care and Use Committee (IACUC) of the Uniformed Services University of the Health Sciences and were conducted in strict accordance with the recommendations of the Guide for the Care and Use of Laboratory Animals [35]. Animals were housed in an environmentally-controlled room approved by the American Association for Accreditation of Laboratory Animal Care (AAALAC). We used the minimum number of animals required to attain statistical significance. No analgesics were administered since non-steroidal anti-inflammatory drugs (NSAIDs) could confound or mask the effect of Stx, and alter the LD50 values. All mice were weighed daily and monitored at least twice per day for morbidity and mortality for two weeks. The animals were checked every 6 h during the time period when mortality was expected. Mortality was an endpoint for the LD_50_ studies, however mice that exhibited signs of extreme morbidity were humanely euthanized by an overdose of inhalational isoflurane followed by cervical dislocation, in accordance with the American Veterinary Medical Association (AVMA) Guidelines on Euthanasia. Extreme morbidity was defined as two or more of the following symptoms: ≥25% weight loss, ruffled fur, lethargy, labored breathing, hunched posture, inability to remain upright, or impaired ambulation that prevented the animal from reaching food and water. The reason we used two signs for extreme morbidity rather than a single symptom, was that mice that exhibit a single sign (usually weight loss) often recover. These mouse LD_50_ studies were reviewed and mortality as an endpoint was approved by the IACUC.

### 3.4. Mouse Toxicity Assay

Six-week-old CD-1 male mice were administered different amounts of Stx2c/c, Stx2d/d, Stx2c/d, Stx2d/c, or 1X PBS intraperitoneally (IP). Five independent experiments were combined to determine the LD_50_ value with a total of 20 control mice and 299 intoxicated mice. Toxin doses ranged from 0.25 ng to 4.1 µg. Mice were monitored for morbidity and mortality for 10 days post-intoxication. The cause of death in the mice that die after injection with Stx is acute tubular necrosis [36]. Probit regression analyses were done with SPSS (IBM Corp., Armonk, NY, USA) to determine the LD_50_ values for the various toxin groups.

### 3.5. Thermostability Assay

Aliquots of each toxin were diluted 1:10 (*v*:*v*) in a total of 50 µL of 1X PBS and exposed to 70 °C for 0, 15, 30, 45, or 60 min and then placed on ice for at least 10 min. The amount of toxin at the start of the assay ranged from 0.2 to 0.7 μg. Samples were then tested for activity by cytotoxicity assay. Linear regression with concomitant statistical analysis was conducted with GraphPad Prism 6 (GraphPad Software, Inc., La Jolla, CA, USA).

### 3.6. Gb3 ELISA

Purified Gb3 (Matreya, Pleasant Gap, PA, USA) was diluted in chloroform/methanol (2:1), and 1 µg was dispensed into each well of a 96-well, *U*-bottom, polypropylene plate. The chloroform/methanol was allowed to evaporate for 3 h. Wells were blocked with 5% BSA in 1X PBS for 1 h at 37 °C and then washed three times with 0.2 mL of 1X PBS + 0.05% Tween 20. Toxins were incubated with 10 ng of the monoclonal anti-Stx2a A subunit antibody 11E10 [37] in 1X PBS at 37 °C for 1 h in an 1.5 mL centrifuge tube, and then two-fold dilutions of these toxins plus antibody were added to the Gb3-coated wells to a total volume of 0.1 mL. Plates were then incubated for 1 h at 37 °C. Wells were washed three times with 0.2 mL 1X PBS + 0.05% Tween 20 before the addition of 0.1 mL of goat anti-mouse-horseradish peroxidase (HRP) diluted 1:2000 in 1X PBS (BD Biosciences, San Jose, CA, USA). The plates were incubated for an additional 1 h at 37 °C, and wells were then washed five times in 1X PBS + 0.05% Tween 20. Next, 0.1 mL of 3,3′,5,5′-tetramethylbenzidine (TMB) peroxidase substrate solution (Bio-Rad) was added to each well, the plates were incubated for 15 min at room temperature, and the peroxidase reaction was stopped by addition of 0.1 mL 1 M H3PO4 to each well. Well contents were transferred to a 96-well clear bottom plate, and the color intensity of each well at A450 was measured to quantify toxin binding to Gb3. The ELISA was done in triplicate, and the apparent *K_d_* values were calculated by application of the GraphPad Prism 6 “One site—Specific binding” algorithm.

### 3.7. Far-UV CD Spectroscopy of Stx2c/c and Stx2d/d

Purified toxins were concentrated by a two-step spin filtration process with Amicon centrifugal filter units (EMD Millipore, Billerica, MA, USA) as follows. First, toxins were placed in a concentrator of pore size 10 kDa and filtered per the manufacturer’s instructions. The retentate was with then placed in a concentrator of pore size 150 kDa and filtered per the manufacturer’s instructions. The flow-through, which contained the purified and concentrated toxins, was dialyzed in 1X PBS with Slide-A-Lyser dialysis cassettes (10K MWCO) and then dialyzed again into 0.1X PBS. Toxins were stored in 0.1X PBS at 4 °C. The concentration of the toxin preparations after these steps was 217 μg/mL (Stx2c) or 163 μg/mL (Stx2d). No aggregation of the proteins was observed after the concentration step or during the CD experiments described below.

Toxins were transferred into quartz cuvettes with path lengths of 1 mm and far-UV CD spectra were recorded with a JASCO J-815 spectrometer (JASCO, Easton, MD, USA) at a scan speed of 20 nm/min and with a bandwidth of 1 nm. After recording an initial spectrum at 20 °C (the average of three sequential spectra), samples were heated at a rate of 1 °C/min to 90 °C and then cooled at 1 °C/min down to 40 °C before recording another CD spectrum (temperature scanning experiments indicated that the unfolding/folding transition was >50 °C for both toxins). The mean residue molar ellipticity, [θ], was calculated as [θ] = (θ × MR)/(10 × *L* × *c*). θ is the measured ellipticity in millidegrees, MR is the mean residue mass (molecular weight of the protein divided by the number of amino acid residues), L is the optical path length in cm and *c* is the protein concentration in mg/mL. Spectral deconvolution was determined with the program available at the online CD spectral analysis suite DichroWeb [38].

## References

[1] Strockbine N.A., Marques L.R., Newland J.W., Smith H.W., Holmes R.K., O’Brien A.D. (1986). Two toxin-converting phages from *Escherichia coli* O157:H7 strain 933 encode antigenically distinct toxins with similar biologic activities. Infect. Immun..

[2] Mead P.S., Griffin P.M. (1998). *Escherichia coli* O157:H7. Lancet.

[3] Richardson S.E., Karmali M.A., Becker L.E., Smith C.R. (1988). The histopathology of the hemolytic uremic syndrome associated with verocytotoxin-producing *Escherichia coli* infections. Hum. Pathol..

[4] Karmali M.A., Petric M., Lim C., Fleming P.C., Arbus G.S., Lior H. (1985). The association between idiopathic hemolytic uremic syndrome and infection by verotoxin-producing *Escherichia coli*. J. Infect. Dis..

[5] Rangel J.M., Sparling P.H., Crowe C., Griffin P.M., Swerdlow D.L. (2005). Epidemiology of *Escherichia coli* O157:H7 outbreaks, United States, 1982–2002. Emerg Infect. Dis..

[6] Mayer C.L., Leibowitz C.S., Kurosawa S., Stearns-Kurosawa D.J. (2012). Shiga toxins and the pathophysiology of hemolytic uremic syndrome in humans and animals. Toxins.

[7] Tarr P.I., Gordon C.A., Chandler W.L. (2005). Shiga-toxin-producing *Escherichia coli* and haemolytic uraemic syndrome. Lancet.

[8] Scheiring J., Andreoli S.P., Zimmerhackl L.B. (2008). Treatment and outcome of Shiga-toxin-associated hemolytic uremic syndrome (HUS). Pediatr. Nephrol..

[9] McGannon C.M., Fuller C.A., Weiss A.A. (2010). Different classes of antibiotics differentially influence Shiga toxin production. Antimicrob. Agents Chemother..

[10] Zhang X., McDaniel A.D., Wolf L.E., Keusch G.T., Waldor M.K., Acheson D.W. (2000). Quinolone antibiotics induce Shiga toxin-encoding bacteriophages, toxin production, and death in mice. J. Infect. Dis..

[11] Bielaszewska M., Idelevich E.A., Zhang W., Bauwens A., Schaumburg F., Mellmann A., Peters G., Karch H. (2012). Effects of antibiotics on Shiga toxin 2 production and bacteriophage induction by epidemic *Escherichia coli* O104:H4 strain. Antimicrob. Agents Chemother..

[12] Wong C.S., Mooney J.C., Brandt J.R., Staples A.O., Jelacic S., Boster D.R., Watkins S.L., Tarr P.I. (2012). Risk factors for the hemolytic uremic syndrome in children infected with *Escherichia coli* O157:H7: A multivariable analysis. Clin. Infect. Dis.: Off. Publ. Infect. Dis. Soc. Am..

[13] Fraser M.E., Fujinaga M., Cherney M.M., Melton-Celsa A.R., Twiddy E.M., O’Brien A.D., James M.N. (2004). Structure of Shiga toxin type 2 (Stx2) from *Escherichia coli* O157:H7. J. Biol. Chem..

[14] Fraser M.E., Chernaia M.M., Kozlov Y.V., James M.N. (1994). Crystal structure of the holotoxin from *Shigella dysenteriae* at 2.5 a resolution. Nat. Struct. Biol..

[15] Jacewicz M., Clausen H., Nudelman E., Donohue-Rolfe A., Keusch G.T. (1986). Pathogenesis of *Shigella diarrhea*. Xi. Isolation of a Shigella toxin-binding glycolipid from rabbit jejunum and hela cells and its identification as globotriaosylceramide. J. Exp. Med..

[16] Lindberg A.A., Brown J.E., Stromberg N., Westling-Ryd M., Schultz J.E., Karlsson K.A. (1987). Identification of the carbohydrate receptor for Shiga toxin produced by *Shigella dysenteriae* type 1. J. Biol. Chem..

[17] Waddell T., Head S., Petric M., Cohen A., Lingwood C. (1988). Globotriosyl ceramide is specifically recognized by the *Escherichia coli* verocytotoxin 2. Biochem. Biophys. Res. Commun..

[18] Muthing J., Meisen I., Zhang W., Bielaszewska M., Mormann M., Bauerfeind R., Schmidt M.A., Friedrich A.W., Karch H. (2012). Promiscuous Shiga toxin 2e and its intimate relationship to forssman. Glycobiology.

[19] Melton-Celsa A.R., Kokai-Kun J.F., O’Brien A.D. (2002). Activation of Shiga toxin type 2d (Stx2d) by elastase involves cleavage of the *C*-terminal two amino acids of the A2 peptide in the context of the appropriate B pentamer. Mol. Microbiol..

[20] Bunger J.C., Melton-Celsa A.R., O’Brien A.D. (2013). Shiga toxin type 2dact displays increased binding to globotriaosylceramide *in vitro* and increased lethality in mice after activation by elastase. Toxins.

[21] Yosief H.O., Iyer S.S., Weiss A.A. (2013). Binding of pk-trisaccharide analogs of globotriaosylceramide to Shiga toxin variants. Infect. Immun..

[22] Bergan J., Dyve Lingelem A.B., Simm R., Skotland T., Sandvig K. (2012). Shiga toxins. Toxicon: Off. J. Int. Soc. Toxinol..

[23] Melton-Celsa A.R. (2014). Shiga toxin (Stx) classification, structure, and function. Microbiol. Spectr..

[24] Scheutz F., Teel L.D., Beutin L., Pierard D., Buvens G., Karch H., Mellmann A., Caprioli A., Tozzoli R., Morabito S. (2012). Multicenter evaluation of a sequence-based protocol for subtyping Shiga toxins and standardizing Stx nomenclature. J. Clin. Microbiol..

[25] O’Brien A.D., Tesh V.L., Donohue-Rolfe A., Jackson M.P., Olsnes S., Sandvig K., Lindberg A.A., Keusch G.T. (1992). Shiga toxin: Biochemistry, genetics, mode of action, and role in pathogenesis. Curr. Top. Microbiol. Immunol..

[26] Ethelberg S., Olsen K.E., Scheutz F., Jensen C., Schiellerup P., Enberg J., Petersen A.M., Olesen B., Gerner-Smidt P., Molbak K. (2004). Virulence factors for hemolytic uremic syndrome, denmark. Emerg. Infect. Dis..

[27] Siegler R.L., Obrig T.G., Pysher T.J., Tesh V.L., Denkers N.D., Taylor F.B. (2003). Response to Shiga toxin 1 and 2 in a baboon model of hemolytic uremic syndrome. Pediatr. Nephrol..

[28] Persson S., Olsen K.E., Ethelberg S., Scheutz F. (2007). Subtyping method for *Escherichia coli* Shiga toxin (verocytotoxin) 2 variants and correlations to clinical manifestations. J. Clin. Microbiol..

[29] Lindgren S.W., Samuel J.E., Schmitt C.K., O’Brien A.D. (1994). The specific activities of Shiga-like toxin type II (Slt-II) and Slt-II-related toxins of enterohemorrhagic *Escherichia coli* differ when measured by vero cell cytotoxicity but not by mouse lethality. Infect. Immun..

[30] Fuller C.A., Pellino C.A., Flagler M.J., Strasser J.E., Weiss A.A. (2011). Shiga toxin subtypes display dramatic differences in potency. Infect. Immun..

[31] Weinstein D.L., Jackson M.P., Perera L.P., Holmes R.K., O’Brien A.D. (1989). *In vivo* formation of hybrid toxins comprising Shiga toxin and the Shiga-like toxins and role of the B subunit in localization and cytotoxic activity. Infect. Immun..

[32] Russo L.M., Melton-Celsa A.R., Smith M.J., O’Brien A.D. (2014). Comparisons of native Shiga toxins (stxs) type 1 and 2 with chimeric toxins indicate that the source of the binding subunit dictates degree of toxicity. PLoS ONE.

[33] Bielaszewska M., Friedrich A.W., Aldick T., Schurk-Bulgrin R., Karch H. (2006). Shiga toxin activatable by intestinal mucus in *Escherichia coli* isolated from humans: Predictor for a severe clinical outcome. Clin. Infect. Dis.: Off. Publ. Infect. Dis. Soc. Am..

[34] Iyoda S., Manning S.D., Seto K., Kimata K., Isobe J., Etoh Y., Ichihara S., Migita Y., Ogata K., Honda M. (2014). Phylogenetic clades 6 and 8 of enterohemorrhagic *Escherichia coli* O157:H7 with particular Stx subtypes are more frequently found in isolates from hemolytic uremic syndrome patients than from asymptomatic carriers. Open Forum Infect. Dis..

[35] National Research Council Committee for the update of the Guide for care and use of laboratory animals (2011). Guide for The Care and Use Of Laboratory Animals.

[36] Wadolkowski E.A., Sung L.M., Burris J.A., Samuel J.E., O'Brien A.D. (1990). Acute renal tubular necrosis and death of mice orally infected with *Escherichia coli* strains that produce Shiga-like toxin type II. Infect. Immun..

[37] Perera L.P., Marques L.R., O'Brien A.D. (1988). Isolation and characterization of monoclonal antibodies to Shiga-like toxin II of enterohemorrhagic *Escherichia coli* and use of the monoclonal antibodies in a colony enzyme-linked immunosorbent assay. J. Clin. Microbiol..

[38] DichroWeb. http://dichroweb.cryst.bbk.ac.uk/html/home.shtml.

